# A scoping review of medical education research in family medicine

**DOI:** 10.1186/s12909-015-0350-1

**Published:** 2015-04-18

**Authors:** Fiona Webster, Paul Krueger, Heather MacDonald, Douglas Archibald, Deanna Telner, Jessica Bytautas, Cynthia Whitehead

**Affiliations:** 1 Department of Family and Community Medicine, University of Toronto, 500 University Avenue, 5th Floor, M5G 1V7 Toronto, ON Canada; 2 Health and BioSciences, Reference Services, Carlton University, 1125 Colonel By Drive, K1S 5B6 Ottawa, ON Canada; 3 Department of Family Medicine, University of Ottawa, 43 Bruyere Street, Floor 3JB, K1N 5C8 Ottawa, ON Canada; 4 Institute of Health Policy, Management and Evaluation, University of Toronto, 155 College Street, 4th Floor, M5T 3M6 Toronto, ON Canada

**Keywords:** Medical education research, Family medicine, Scoping review

## Abstract

**Background:**

Little is known about the state of education research within family medicine. As family medicine education models develop, it is important to develop an understanding of the current state of this research and develop ways to advance the field.

**Methods:**

We conducted a scoping review of family medicine education research to describe 1) research topic areas and 2) the methodologies and methods used to study these topics. MEDLINE, Social Sciences Abstracts and ERIC electronic databases were searched. 817 full text articles from 2002 to 2012 were screened; 624 articles were included in the review.

**Results:**

The following research topic areas were identified: continuing education, curriculum development, undergraduate education, teaching methods, assessment techniques, selection of entrants, non-clinical skills, professional and faculty development, clinical decision-making and resident well-being. Quantitative studies comprised the large majority of research approaches; overall minimal methodological details were provided.

**Conclusions:**

Our review highlights an overall need for increased sophisticated in methodological approaches to education research in family medicine, a problem that could be ameliorated by multiple strategies including better engagement of methodologists throughout the research process. The results provide guidance for future family medicine education research programs.

## Background

The field of medical education has grown dramatically over the past fifty years, demonstrated by the significant increase in the number of publications, journals and conferences dedicated to this topic [1-3]. As the field develops, stakeholders are concerned with examining its progress, and in particular its success in developing productive programs of research [4-6]. Reviews of medical education research have been valuable in illuminating main thematic areas that have been studied as well as methodological limitations of this work; these findings have led to numerous debates about gaps to be addressed, priority areas to be examined and the criteria that should be used to assess progress [3-8]. These reviews have covered this topic in a broad [3,5] and more targeted approach [9,10], each of which has value in informing medical education research agendas. One area of medical education research that has not yet been systematically examined is family medicine. Given the potentially unique educational foci of family medicine and current investments being made in family medicine education research, this study is a timely addition to the literature.

Family medicine, positioned within the broader domain of primary care, plays a pivotal role in robust and efficient health care systems [11,12]. Medical education needs to address the unique elements of family medicine trainees and practitioners within the broader organizational, social and regulatory contexts in which they work. For example, the work of family physicians is changing with the development and implementation of new primary care models in Canada and internationally [13,14]. In this time of rapid change and increasing demand on community-based primary health care, medical education research is required to ensure that family physicians are trained to meet new and evolving health care challenges and best serve the needs of patients. There have also been developments in educational models including the movement of family medicine training to a competency-based curriculum framework [15]. How these changes impact family medicine trainees’ experience and their knowledge, attitude and skills is important to assess for the future of family medicine.

Academic family medicine departments are increasingly recognizing the importance of educational research and are creating support structures for its advancement. Researchers have advocated for the development of *programs* of research rather than the conduct of single studies [16,17]. Regehr [5] noted that there is a distinction between scholars working on the same topic and scholars working toward a shared goal. The first step in developing a research agenda that is relevant to the field of family medicine is to understand the current state of family medicine education research.

The aim of this scoping review is to examine the volume, topics and methodological nature of research activity in family medicine education research. These findings will inform future education research directions, including programmatic research areas, while also establishing a benchmark to assess changes in educational scholarship over time.

## Methods

A scoping review approach was used for this study. The scoping review is a strategy designed to ‘map’ literature in a particular area, in contrast to a systematic review that more commonly addresses a specific research question. A scoping review illuminates key concepts, and main sources and types of evidence. It is particularly useful for topics that are complex, have not been extensively reviewed and for which many different study designs have been used [18]. A scoping review does not explicitly aim to assess the quality of studies but can identify research gaps in the existing literature. We used the methodologically rigorous scoping review approach proposed by Arksey and O’Malley [18] and advanced by Levac, Colquhoun, and O’Brien [19]. This approach involves five stages: (1) identifying the research questions, (2) identifying relevant studies, (3) study selection, (4) charting the data and (5) collating, summarizing and reporting results.

### Identifying the research question

The research questions for this review were developed in collaboration with the research team, and were outlined as:

1. What research questions/topic areas have been studied in family practice education research?
2. What research designs have been used to study these topics?

### Identifying relevant studies

We searched the electronic databases MEDLINE, Social Sciences Abstracts, and ERIC using the following search terms identified through input from the research team and in consultation with an experienced information specialist (HM): family physician, family practice, general practitioner, primary care physician, and community practice. Using the term “AND”, these were combined with the terms: medical education, curriculum, learning, and teaching. Both medical subject headings (MeSH) and free text terms were used. To supplement the search, we scanned the reference lists of included studies and searched the authors’ personal files. In addition, we drew on the extensive networks of our review team to contact people who are leaders in family medicine education research to vet our search and identity missing publications. The team information specialist executed all final searches, exported the results into RefWorks, and removed all duplicates from the search results.

### Study selection

We included studies that used quantitative, qualitative or mixed methods designs; focused exclusively on family medicine trainees and practitioners and/or primary care; had an explicit focus on medical education; were conceptualized as research or evaluation work; and were written in the English language. We used the following definition of medical education:

“A broad definition of medical education research would include any investigation related to the education of medical professionals, including research related to undergraduate (medical school), graduate (residency), and continuing medical education. Medical education research can focus on any number of topics, including curriculum development, teaching methods, student evaluation, teacher evaluation, course evaluation, faculty development, admission and preparation of candidates for medical training, factors influencing career choice, research methodology, and use of technology in education”. ([20], p. 640)

We excluded papers that focused on primary care by other generalist groups (e.g. internal medicine, pediatrics) and professions (e.g. nurse practitioners) and interprofessional education, which were beyond the scope of the current study. We also excluded commentaries and papers solely focused on descriptions of curriculum development given the lack of a research component. We did not use methodological quality criteria in our study selection.

Prior to commencing the screening process, a calibration exercise was conducted to ensure reliability in correctly selecting articles for inclusion. This entailed screening a random sample of 5% of the included citations by two reviewers, independently. Eligibility criteria were modified if low agreement (e.g., a kappa statistic less than 80%) was observed between the reviewers. The reviewers then independently screened the remainder of the search results for inclusion using a pre-defined relevance criteria form for all levels of screening (e.g., title and abstract, full-text review). Discrepancies were resolved by consensus or the involvement of a third reviewer.

### Charting the data

A data abstraction form was drafted and tested independently by two reviewers on a random sample of 10 articles and revised iteratively by the study team. The final form included the following general headings: study characteristics (e.g. year of publication, country where research was conducted, sample size); research topic area, and methodology and methods employed. Members of the research team independently read each article and extracted the relevant data. Any uncertainty regarding abstraction was resolved by discussion with another research team member or the involvement of a second reviewer. We did not formally appraise methodological quality because the aim of a scoping review is to identify gaps in the evidence base and to target topic areas for future reviews.

### Collating, summarizing and reporting results

We synthesized the data according to topic areas of medical education research conducted and research designs used. Data analysis involved quantitative frequency analysis and qualitative thematic analysis. The results are reported below in tabular and narrative forms.

## Results

**Figure 1:**
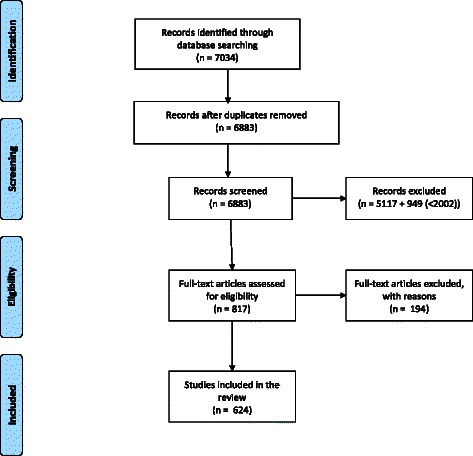
Flow chart of search results.

An initial search in February 2012 retrieved over 6,000 citations. After screening titles and abstracts, there were 1725 eligible citations based on our inclusion/ exclusion criteria. At this point we made the decision to restrict our search to a 10-year period for reasons of feasibility. 817 full text articles from 2002 to 2012 were screened. For 79 articles, full text was not available. 109 more articles did not meet the inclusion criteria and 6 duplicates were identified. 624 articles were included in the review (see Figure 1).

### Descriptive results

Studies originated from 40 different countries. Just over half of the studies were based in North America (55%, 343), the majority in the United States of America (274). European studies comprised 29% (179) of the studies, with the majority of being conducted in the United Kingdom. The remaining studies were undertaken in Australia/Oceania (8%, 50), Middle East/North Africa/Greater Arabia (5%, 29), Asia (3%, 15), Central/South America (0.3%, 2) and Sub-Saharan Africa (0.3%, 2).

Studies were published in 138 different journals, although 62% (385) of the studies were concentrated in ten journals. These included 42% (162) of studies published in “Family Medicine”, 12%, (46) in “Medical Education”, 11% (43) in “Medical Teacher” and 11% (40) in “Academic Medicine”.

### Research topic areas

The largest percentage of studies focused on the topic of continuing education (259, 37%). Continuing education includes the “educational activities that serve to maintain, develop, or increase the knowledge, skills, and professional performance and relationships a physician uses to provide services for patients, the public, or the profession” ([21], p. 10S). Curriculum development, undergraduate education, teaching methods, and assessment techniques each composed about 10% of the research topics (11%, 79; 10%, 71; 10%, 69; 9%, 66; respectively). Selection of entrants for family medicine training and non-clinical skills composed 7% (49) and 6% (41), respectively. Professional development, faculty development, and clinical decision-making composed about 2% of the topics (19, 13, 8, respectively). Only one study focused on the topic of resident well-being (see Table 1 for research topic areas).

**Table 1:** **Research topic areas**

Research topic area	Percent (number) of papers
Continuing education	37% (259)
Curriculum development	11% (79)
Undergraduate education	10% (71)
Teaching methods	10% (69)
Assessment techniques	9% (66)
Selection of entrants	7% (49)
Non-clinical skills	6% (41)
Training of teachers	4% (25)
Professional development	3% (19)
Faculty development	2% (13)
Clinical decision making	1% (8)
Resident well-being	0% (1)

### Research approach

Across all topic areas, the majority of studies (75%, 484) used a quantitative research approach. The remaining used a qualitative (15%, 100) or a mixed-methods (6%, 36) approach; 4% (25) of the studies did not have an explicit approach. The number of publications using a qualitative research approach increased overall between 2002 and 2011 from 8 to 17, although the growth during this time period was not steady.

The majority of quantitative studies (65%, 315) and qualitative studies (86%, 86) did not explicitly identify the methodology used. Of the quantitative studies that did indicate a methodology, 23% (113) used pre/post tests, 10% (47) used a randomized controlled trial, and retrospective and prospective cohort studies composed 1% each (7 and 3, respectively). Of the qualitative studies, 8% (8) were identified as grounded theory and 6% (6) as phenomenology.

The most commonly used methods across research topic areas were documentation (i.e., medical records, exam/test results) and surveys. For the studies with no specified methodology, nearly half employed surveys as their primary method of data collection (49%, 203). Documentation (i.e., medical records, exam/test results) was used in 31% (131) of the studies, followed by interviews (10%, 40), observation (6%, 23) and focus groups (3%, 14). The primary method of data collection was not specified in 5 studies (1%). The majority of studies with unspecified methodologies were described as evaluation work (69%, 287), with the remainder (29%, 122) reported as research (122).

## Discussion

We identified 624 articles published between 2002 and 2012 on family medicine education research. This large number of studies indicates that the field is developing and plays an important role in the broader field of medical education. However, the limitations in methodological reporting and the narrow range of methodologies and methods used, demonstrates the need for better reporting in family medicine education research and may be a proxy for rigour in some cases. These findings provide a benchmark for to further develop and strengthen family medicine education research.

The most frequently studied topic area in family medicine education research during the time period examined was continuing education. This finding contrasts with other reviews of the broader medical education field, which have tended to focus on undergraduate medical curriculum [6], in particular student assessment, clinical and communication skills, clinical clerkships and problem based learning [2]. More recently, the broader medical education research field has studied topics of professionalism, patient safety, scholarship in education, and the role of humanities in medical education [2]. It would be valuable to explore whether further alignment between family medicine education research and the broader medical education field might benefit the discipline. Family medicine education researchers should also consider how their research corresponds to recently identified priority areas of medical education, particularly those of relevance to family medicine such as addressing individual and community needs, promoting prevention, diversity of learning contexts and valuing generalism [22].

The vast majority of studies in this review, irrespective of topic area, used a quantitative design. A recent review by Lee et al. of the medical education literature reported similar findings [3]. Medical education researchers have noted that work in this area is largely ‘effectiveness’ rather than ‘discovery’ driven, and there is a need for studies that ask ‘how and why does it work’ [2,7]. Qualitative methodologies are particularly well suited to understanding the ‘hows’ and ‘whys’ of a given phenomenon and have gained considerable traction and recognition within the health sciences [23]. Qualitative studies can provide a much deeper understanding of individuals’ perspectives and experiences and the influence of social processes in medical education. The choice of a quantitative or qualitative research design is dependent on the particular research question, yet both approaches should play key roles in the field given the complexity of the topic of medical education research. Although we saw an increase in the number of qualitative studies published in family medicine education research between 2002 and 2012, we advocate for a greater methodological breadth, including the use of critical qualitative methodologies.

We identified a remarkably large percentage of quantitative and qualitative studies for which no specific methodology was available. A methodology describes the process or design lying behind the choice and use of particular methods and links the choice and use of methods to the desired outcomes [24]. This limitation reflects the broader field of medical education research, where researchers have expressed concern about the lack of details about research paradigm or epistemological assumptions underpinning work conducted [25] as well as lack of quality reporting of experimental studies [8]. As Bunniss and Kelly [25] note, articulation of underlying assumptions is needed if researchers are to critically engage with study findings. This further highlights the need to incorporate more theoretical frameworks into family medicine education research. Furthermore, medical education research largely focuses on the individual student and his/her learning; other perspectives that could be used include systems, sociological, economic and ecological perspectives [2].

Within the subset of studies for which no specific methodology was available, the large majority was classified as evaluation studies. Whereas research aims to contribute to fundamental knowledge and theory and to illuminate societal concerns, evaluation aims to determine or improve program effectiveness [26].

There is some concern that evaluation studies are not as rigorous as research studies [27], which may help to account for the apparent lack of high quality studies. Furthermore, survey instruments were commonly used as the primary method of data collection. Surveys that rely on self-reporting have limitations as a sole method of data collection. In the field of medical education more broadly, emerging criticism has been that research is often confined to pre-post-test surveys to evaluate specific teaching approaches [28-31]. Both research and evaluation work is needed in the field of family medicine education with standards of quality expected for both.

A limitation of this scoping review is that we did not perform targeted searches for grey literature. This review covers the literature up to the year 2012; however, the authors who are actively involved in the field of family medicine education research are not aware of significant change in trends in family medicine education research since that time. This scoping review did not explicitly aim to assess quality of studies included; however important methodological limitations were identified. Given the large number of articles included in the review, it was challenging to provide more detailed information about the particular thematic areas explored. We recommend that future reviews examine specific topic areas within the broader umbrella of family medicine education research in more depth.

## Conclusion

This scoping review provides an important high-level synthesis of the field of family medicine education research. In particular, it highlights an overall need for increased sophisticated in methodological approaches to education research in this field. We therefore propose that efforts to strengthen the field should attend to the following issues. The methodological rigour and diversity of family medicine education research needs attention; this problem could be ameliorated by better engagement of methodologists throughout the research process. In addition, research should be explicitly theoretically informed; this can be achieved by engaging scholars with diverse areas of theoretical expertise. Researchers in family medicine education research should work together in identifying current and relevant research themes, those that are unique to family medicine as well as those that are relevant to the larger field of medical education research. Working collaboratively with researchers in other fields such as continuing education, interprofessional education and patient safety can support these researchers’ intersecting areas of interest. A breadth of research topics is positive but a community of researchers should also aim for an integration of work into coherent programmatic efforts that can advance the field [5].
